# Orexin Deficiency in Narcolepsy: Molecular Mechanisms, Clinical Phenotypes, and Emerging Therapeutic Frontiers

**DOI:** 10.1002/brb3.70984

**Published:** 2025-10-11

**Authors:** Rameesha Rauf, Salwa Asif, Abdallah AlSaafeen, Kaviprada Geathaa Dhantapaani, Maryam Muhammad Nadeem, Zohra Kamran Rao, Aliya Shaju Shahul Hameed, Karthik Chintharala, Muhammad Faheem Anwar, Asad Ali Ahmed Cheema

**Affiliations:** ^1^ Tbilisi State Medical University Tbilisi Georgia; ^2^ EUMD, General Medicine Tbilisi State Medical University Tbilisi Georgia; ^3^ Nantong Medical University Nantong City China; ^4^ Tbilisi State University Tbilisi Georgia; ^5^ East European University Tbilisi Georgia; ^6^ NRI Academy of Medical Sciences Guntur India; ^7^ International School of Medicine International University of Kyrgyzstan Bishkek Kyrgyzstan

**Keywords:** autoimmune neurodegeneration, hypocretin, narcolepsy Type 1, orexin deficiency, sleep–wake regulation, therapeutic targets

## Abstract

**Introduction:**

Narcolepsy Type 1 (NT1) is a chronic neurological disorder characterized by excessive daytime sleepiness (EDS), cataplexy, and REM intrusions, caused by a deficiency of orexin (hypocretin), a hypothalamic neuropeptide essential for arousal, REM sleep regulation, metabolism, and emotional stability. This review synthesizes and critically analyzes the pathophysiological, clinical, and therapeutic dimensions of orexin deficiency in narcolepsy, with particular emphasis on recent advances from 2023 to 2025.

**Methods:**

This narrative analytical review analyzed 50 peer‐reviewed publications covering the neurobiology of the orexin system, diagnostic evolution of NT1, comparative symptomatology of central hypersomnia disorders, and recent therapeutic innovations. Out of which 16 studies were selected based on relevance, recency, and translational impact.

**Results:**

Recent research confirms that over 90% of NT1 patients exhibit cerebrospinal fluid (CSF) orexin‐A levels below 110 pg/mL and carry the HLA‐DQB1*06:02 allele, indicating a strong genetic and immunological association. Postmortem analyses have revealed a loss of up to 95% of orexin‐producing neurons in the lateral hypothalamus. In a 2024 multicenter trial, danavorexton (a selective orexin receptor‐2 [OX2R] agonist) was associated with a mean improvement of 11.1 points on the Maintenance of Wakefulness Test, outperforming modafinil and suggesting the feasibility of receptor‐level restoration. In addition, intermediate orexin levels (110–200 pg/mL) have been documented in a subset of patients with narcolepsy Type 2 (NT2) and idiopathic hypersomnia (IH), challenging the binary diagnostic threshold and prompting reevaluation of orexin's diagnostic role. Orexin dysfunction has also been correlated with high psychiatric comorbidity rates, including major depressive disorder in 40% and anxiety disorders in nearly 30% of NT1 patients. These manifestations reflect orexin's broader role in regulating REM sleep, metabolic processes, and autonomic stability.

**Conclusion:**

Orexin deficiency remains the central mechanism underlying NT1, with far‐reaching implications in psychiatric and neurodegenerative disease. While emerging orexin‐targeted therapies offer promising disease‐modifying potential, critical challenges persist in standardizing biomarkers, resolving NT2 classification ambiguities, and ensuring global access to therapeutics.

## Graphical abstract

Orexin deficiency drives narcolepsy Type 1, leading to excessive daytime sleepiness, cataplexy, and REM intrusions. This review highlights recent advances (2023–2025) in molecular mechanisms, diagnostics, and emerging orexin‐targeted therapies with the potential for disease modification.

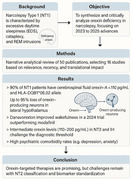

## 1 Introduction

Orexins, also known as hypocretins, are neuropeptides produced by a small group of neurons in the lateral hypothalamus, first identified in 1998 (Williams et al. 2011). These neuropeptides orexin‐A and orexin‐B bind to two G‐protein‐coupled receptors, OX1R and OX2R, which are widely distributed across the brain, particularly in arousal‐related regions like the locus coeruleus, tuberomammillary nucleus, and ventral tegmental area (VTA) (Khairuddin et al. 2020). Orexin signaling plays a critical role in stabilizing the sleep–wake cycle, maintaining alertness, suppressing inappropriate transitions into REM sleep, and integrating behavioral states such as stress, hunger, and motivation (AJMC 2024).

In healthy individuals, orexin levels decline at night to allow regular REM sleep, but remain sufficiently active to prevent fragmentation and atonia during wakefulness (Saper et al. 2005). Conversely, pharmacological inhibition of orexin receptors, such as with dual orexin receptor antagonists (DORAs), can induce sleep without abolishing REM, illustrating the role of orexin in modulating REM (Barateau et al. 2020).

Narcolepsy is a chronic neurological disorder that affects approximately 1 in every 2000 people globally, with NT1 accounting for nearly 70% of cases (Berridge et al. 2010; Nishino et al. 2010). Narcolepsy Type 1 (NT1) is characterized by excessive daytime sleepiness (EDS), cataplexy, hypnagogic hallucinations, sleep paralysis, and disrupted nocturnal sleep. The central pathophysiology of NT1 is characterized by the selective loss of orexin‐producing neurons in the lateral hypothalamus (Biscarini et al. 2025). This loss results in orexin concentrations in cerebrospinal fluid (CSF) falling below 110 pg/mL, a diagnostic hallmark of the disorder (Broughton and Mamelak 1979; Nishino et al. 2000). Studies confirm that up to 95% of NT1 patients carry the HLA‐DQB1*06:02 allele, strongly suggesting an autoimmune etiology, though no specific autoantibody has been universally identified (Cureus Review Panel 2023; Dauvilliers et al. 2023).

The role of orexin was further established in animal models, where genetic ablation of orexin neurons reproduced the complete narcoleptic phenotype, including sleep‐onset REM episodes and emotion‐triggered cataplexy (Dauvilliers et al. 2009). Recent research from 2023 to 2025 has revealed that a subset of orexin neurons continues to function during REM sleep and inhibits further REM re‐entry; their loss is directly linked to increased REM density, dream intrusions, and cataplexy‐like phenomena (de Lecea and Huerta 2023; de Lecea et al. 1998). Moreover, orexin's regulatory scope now extends beyond sleep, encompassing thermoregulation, cardiovascular control, metabolic homeostasis, and affective behavior, making its deficiency relevant to a wide array of psychiatric and neurological disorders (E. Mignot 2001; Esmaili‐Shahzade‐Ali‐Akbari et al. 2024).

The therapeutic landscape has evolved significantly. Traditional wake‐promoting agents like modafinil and amphetamines offer symptomatic relief but do not address the underlying pathophysiology. In contrast, novel orexin receptor agonists such as danavorexton (TAK‐925) and ALKS 2680 aim to restore orexin function at the receptor level. Preliminary trials have demonstrated improvements in sleep latency, vigilance, and REM control, although safety concerns, such as hepatotoxicity, remain an ongoing challenge (Stefani and Högl 2020; Nollet and Leman 2013).

This review critically examines the role of orexin deficiency in narcolepsy from a neurobiological, clinical, and therapeutic perspective. It integrates current research on the molecular mechanisms of orexin signaling, explores the evidence supporting immune‐mediated neuron loss in NT1, and analyzes how orexin dysfunction correlates with the clinical phenotype. It further evaluates the broader implications of orexin deficiency in neuropsychiatric and neurodegenerative disorders, reviews existing and emerging treatment options, and highlights key controversies and research gaps. Specifically, this review seeks to answer the question: To what extent does orexin deficiency account for the pathophysiology of narcolepsy, and can therapies that restore or mimic orexin function effectively reverse narcoleptic symptoms?

## 2 Methodology

This narrative review was conducted in accordance with PRISMA‐Narrative guidelines. A targeted literature search was performed across PubMed, Scopus, Web of Science, and ScienceDirect, focusing on publications from 2020 to 2025. Search terms included combinations of “orexin,” “narcolepsy,” “hypocretin,” “NT1,” “NT2,” “REM intrusion,” and “orexin receptor agonists.” From an initial pool of 50 peer‐reviewed articles, 16 were selected for full thematic inclusion based on clinical relevance, methodological rigor, and translational impact. Studies addressing orexin signaling, deficiency‐related pathophysiology, diagnostic criteria, and emerging therapies were prioritized. Findings were synthesized across five key domains: neurobiology, clinical symptomatology, diagnostics, treatment advances, and broader neuropsychiatric or metabolic implications. Quantitative meta‐analysis was not performed due to the narrative nature of the review.

## 3 Results

To comprehensively evaluate the multidimensional impact of orexin deficiency in narcolepsy, a curated selection of 14 high‐impact studies published between 2020 and 2025 was analyzed. These publications encompass a diverse range of methodologies, including animal models, human clinical trials, immunopathological investigations, and therapeutic assessments. Table 1 presents a synthesized overview of each study's author(s), type, and primary findings. Together, these studies underscore the critical role of orexin in stabilizing REM architecture, regulating autonomic function, mediating emotional processing, and informing the therapeutic transition from symptomatic agents to receptor‐level orexin restoration. This evidence base forms the backbone of the current narrative analytical review.

**TABLE 1:** Summary of selected peer‐reviewed studies (2020–2025) examining orexin's role in narcolepsy pathophysiology, clinical features, and therapeutic innovation. Studies include original research, reviews, and case reports that collectively contribute to the evolving understanding of orexin deficiency and its clinical implications in NT1 and related central hypersomnia disorders.

Author	Study	Findings
Mogavero et al. (2023)	Review	Orexin's primary role may be to suppress REM sleep rather than simply promote wakefulness, as shown by its highest levels at sleep onset and lowest levels during natural morning awakening when REM peaks.
H. Ito et al. (2023)	Original Research	Mice lacking orexin receptors specifically during sleep exhibited fragmented and unstable REM sleep episodes, demonstrating that orexin signaling while asleep is essential for maintaining consolidated REM sleep architecture.
Zhang and Khatami (2025)	Original Research	Patients with narcolepsy showed normal or heightened physiological stress markers despite reporting lower subjective stress levels, suggesting that orexin deficiency disrupts the connection between perceived and bodily stress responses.
Mignot et al. (2021)	Review	Hypocretin/orexin deficiency in narcolepsy leads not only to excessive daytime sleepiness but also to fragmented nighttime sleep, underscoring that orexin plays a vital role in maintaining stable and consolidated sleep across the night.
Wasserman et al. (2020)	Case Report	A rare case where cataplexy symptoms resolved despite persistent orexin deficiency, indicating that cataplexy can improve independently of orexin levels.
Grandner et al. (2025)	Review	The chronic sleep fragmentation and autonomic dysfunction seen in narcolepsy contribute to heightened cardiovascular risk by promoting inflammation, metabolic disturbances, and impaired blood pressure regulation.
Straat et al. 2000	Review	Type 1 narcolepsy develops primarily due to the loss of orexin‐producing neurons in the hypothalamus, which disrupts sleep‐wake regulation and leads to excessive daytime sleepiness and cataplexy.
Mațotă et al. (2023)	Review	Narcolepsy is characterized by a complex disruption of the sleep‐wake cycle, involving both excessive daytime sleepiness and abnormal REM sleep regulation, which significantly impairs patients’ daily functioning.
Dauvilliers et al. (2023)	Review	Oral orexin 2 receptor agonist TAK‐994 (firazorexton) which selectively activates the OX_2_ receptor significantly improved symptoms in narcolepsy type 1 patients to the extent that many reported feeling effectively cured.
Berteotti et al. (2021)	Review	Alterations in orexin signaling contribute to widespread neurological dysfunction by disrupting the balance of arousal and sleep regulation, which in turn impacts cognitive processes and may exacerbate symptoms in disorders such as Alzheimer's, Parkinson's, and epilepsy.
Pizza et al. (2022)	Review	The loss or disruption of orexin signaling leads to instability in the sleep‐wake cycle by impairing the brain's ability to maintain consistent wakefulness and suppress inappropriate transitions into REM sleep, which contributes to sleep disorders like narcolepsy.
Biscarini et al. (2024)	Review	Abnormalities in orexin signaling critically disrupt the regulation of REM sleep, leading to the characteristic symptoms of narcolepsy such as excessive daytime sleepiness and cataplexy.
Thomaz et al. (2024)	Review	Orexin replacement therapies help restore normal sleep‐wake regulation by compensating for the loss of orexin‐producing neurons, leading to improved wakefulness and decreased cataplexy episodes in narcolepsy type 1 patients.
Xu et al. (2024)	Review	Autoreactive T cells play a crucial role in the development of narcolepsy type 1 by targeting and destroying orexin‐producing neurons, supporting the autoimmune hypothesis of the disease's pathogenesis.

## 4 Discussion

### 4.1 The Orexin System: Molecular and Neurophysiological Perspective

Orexins are excitatory neuropeptides synthesized in the lateral hypothalamus. The precursor, prepro‐orexin, is post‐translationally cleaved into orexin‐A and orexin‐B, which act via two G‐protein‐coupled receptors: orexin Receptor 1 (OX1R) and orexin Receptor 2 (OX2R) (Greer et al. 2017). OX1R exhibits greater affinity for orexin‐A, while OX2R binds both peptides with similar affinity (Khairuddin et al. 2020). These receptors are differentially distributed. OX1R is primarily found in the locus coeruleus and VTA. At the same time, OX2R localizes to the tuberomammillary nucleus and dorsal raphe, regions essential to arousal and REM suppression (AJMC 2024).

Orexin‐producing neurons have widespread projections across the brain, including the cortex, thalamus, basal forebrain, and brainstem. This anatomical distribution enables them to orchestrate diverse physiological functions, including wakefulness, feeding behavior, reward processing, stress adaptation, and thermoregulation (Bunney et al. 2017; Sato and Sakurai 2023). Notably, these neurons are tonically active during wakefulness and silent during REM and non‐REM sleep, indicating their role in stabilizing vigilance states (Berridge et al. 2010).

The orexin system is a major regulator of sleep‐wake stability. Through excitatory projections to monoaminergic and cholinergic nuclei such as the locus coeruleus (noradrenergic), raphe nuclei (serotonergic), and basal forebrain (cholinergic), orexins help sustain cortical arousal (Ito et al. 2023; Liguori 2016). Simultaneously, orexins inhibit REM‐generating regions in the brainstem, such as the sublaterodorsal nucleus, thus suppressing REM sleep intrusions into wakefulness (Singh and Biswas 2023). This dual action explains why loss of orexin signaling in NT1 leads to both EDS and REM‐related phenomena, such as cataplexy and sleep‐onset REM periods (SOREMPs) (Cureus Review Panel 2023). Recent optogenetic and pharmacological studies further support this model. In orexin‐deficient mice, the loss of inhibitory control over REM circuit results in increased REM bouts and REM‐related atonia during wakefulness, resembling cataplexy (Han et al. 2020). These findings confirm that the orexin system serves as a switch that regulates wakefulness and controls the transition to REM sleep.

Orexin signaling extends beyond sleep regulation into several critical domains:

- *Metabolic regulation*: Orexin neurons are responsive to peripheral signals, including glucose, leptin, and ghrelin. They are activated during fasting and promote feeding behavior, energy expenditure, and thermogenesis (Dauvilliers et al. 2009). Orexin‐deficient individuals often exhibit weight gain despite reduced food intake, possibly due to metabolic dysregulation (de Lecea and Huerta 2023).
- *Autonomic control*: Orexins modulate cardiovascular and respiratory functions via sympathetic pathways. NT1 patients exhibit blunted heart rate variability, reduced nocturnal blood pressure dipping, and impaired thermoregulation, reflecting the role of orexin in autonomic homeostasis (Lang et al. 2004; Lawrence et al. 2011; Han et al. 2020).
- *Affective regulation*: Orexinergic projections to the amygdala, prefrontal cortex, and nucleus accumbens influence emotional behavior and reward processing. Dysregulated orexin signaling has been implicated in anxiety, depression, and addiction comorbidities frequently seen in narcoleptic patients (E. Mignot 2001; Esmaili‐Shahzade‐Ali‐Akbari et al. 2024; Mahoney et al. 2023).

The pleiotropic role of orexins complicates the interpretation of their deficiency. While their role in narcolepsy is unequivocal, emerging evidence suggests involvement in diverse disorders such as major depressive disorder (MDD), Parkinson's disease (PD), and even Alzheimer's dementia (Maaz et al. 2025; Stefani and Högl 2020). This broader perspective challenges the notion of orexin as a sleep‐specific molecule and opens avenues for its therapeutic targeting in psychiatry and neurology. For instance, DORAs (e.g., suvorexant) used in insomnia may alter mood circuits and produce residual sedation in some individuals (Nollet and Leman 2013). Conversely, selective OX2R agonists like danavorexton appear to restore wakefulness in narcoleptic patients without affecting mood or metabolism adversely (Greer et al. 2017; Medical News Today 2023; Meglio 2023, E. J. Mignot 2014). These differential effects emphasize the need for precision‐targeted orexin therapies.

The orexin system serves as a master integrator of arousal, regulation of REM sleep, metabolism, and emotional processing. Its dysfunction, whether due to deficiency or dysregulation, produces a wide range of physiological and behavioral consequences. Future treatments for narcolepsy and other neuropsychiatric disorders will benefit from exploiting receptor‐specific orexin modulation to restore functional balance with minimal off‐target effects.

### 4.2 Narcolepsy and Orexin Deficiency: An Evolving Paradigm

The understanding of narcolepsy has progressed from early observational descriptions in the 19th century to a robust neurobiological framework in the 21st century. First clinically described by Jean‐Baptiste‐Édouard Gélineau in 1880, narcolepsy was initially viewed as a seizure‐like disorder due to its sudden sleep episodes. However, modern research has firmly established narcolepsy, particularly NT1 as a chronic neurological disorder of central origin, linked to the selective degeneration of orexin‐producing neurons in the lateral hypothalamus (Williams et al. 2011).

Narcolepsy affects an estimated –5 million people worldwide, yet remains underdiagnosed or misdiagnosed in nearly 60% of cases, often as depression, epilepsy, or chronic fatigue syndrome (Khairuddin et al. 2020; Ito et al. 2023). NT1 is the more severe form, characterized by EDS, cataplexy, hypnagogic hallucinations, and REM intrusion phenomena, all of which correlate strongly with CSF orexin‐A concentrations below 110 pg/mL (AJMC 2024). In contrast, narcolepsy Type 2 (NT2) lacks cataplexy and is associated with normal orexin levels, making its pathogenesis more ambiguous (Barateau et al. 2020; Liguori 2016).

The link between narcolepsy and REM dysregulation was first hinted at in 1957 by Yoss and Daly, who identified REM abnormalities through EEG analysis. A breakthrough occurred in 1998, when two groups simultaneously discovered the orexin neuropeptides and their crucial role in regulating sleep and arousal (Berridge et al. 2010). Shortly thereafter, it was demonstrated that narcoleptic dogs and genetically modified mice lacking orexin or its receptors exhibited symptoms identical to human NT1, including cataplexy and sleep‐onset REM episodes (Biscarini et al. 2024; Singh and Biswas 2023).

The first direct evidence of orexin neuron loss in humans was obtained from postmortem hypothalamic studies, which confirmed neuronal depletion of up to 90% in NT1 patients (Broughton and Mamelak 1979). This pivotal finding was further substantiated by consistent CSF orexin‐A deficiency in symptomatic individuals and is now a cornerstone diagnostic marker in clinical sleep medicine. Today, the International Classification of Sleep Disorders (ICSD‐3) and DSM‐5 recognize NT1 and NT2 as distinct subtypes, with orexin deficiency as the defining biomarker for NT1. Table 2 outlines the key historical and diagnostic advances that led to the development of this modern classification paradigm.

**TABLE 2:** Major Milestones in Narcolepsy Research and Classification.

Year	Milestone
1880	The term “narcolepsy” was coined by Gélineau
1957	REM abnormalities observed in narcoleptics (Yoss and Daly)
1998	Discovery of orexins (Sakurai et al.; de Lecea et al.)
1999	Orexin‐deficient knockout mice show narcolepsy symptoms
2000	Postmortem confirmation of orexin neuron loss in humans
2001‐2013	NT1/NT2 distinction formally integrated into ICSD/DSM

In NT1, CSF orexin‐A concentrations are typically < 110 pg/mL, while healthy individuals average > 200 pg/mL (AJMC 2024). This drastic reduction reflects the near‐total loss of the estimated 70,000–80,000 orexin neurons usually present in the hypothalamus (Cureus Review Panel 2023; Lawrence et al. 2011). The loss is particularly adjacent neurons in the same region remain intact, ruling out global hypothalamic degeneration. Neuroimaging studies in 2024 have further confirmed reduced hypothalamic volume and connectivity changes in orexin‐related arousal circuits among NT1 patients, correlating with symptom severity scores (Dauvilliers et al. 2023).

The autoimmune hypothesis posits that NT1 arises from immune‐mediated destruction of orexin‐producing neurons. This view is supported by:

- HLA‐DQB1*06:02 positivity in over 95% of NT1 cases across all ethnic groups—compared to 12%–25% in the general population (Dauvilliers et al. 2009).
- Temporal clustering after infections, especially 2009 H1N1 influenza and Pandemrix vaccination, which were associated with sudden NT1 onset in children and adolescents (de Lecea and Huerta 2023).
- Increased levels of cytotoxic CD8+ T cells, interferon‐gamma, and interleukin‐6 in affected individuals (de Lecea et al. 1998).

However, the absence of consistent orexin‐specific autoantibodies and the lack of direct histological evidence of immune cell infiltration into the hypothalamus weaken the hypothesis. Despite this, T‐cell receptor sequencing in 2025 studies showed clonally expanded autoreactive CD4+ T cells in up to 43% of newly diagnosed NT1 cases, suggesting antigen‐specific responses (E. Mignot 2001).

Furthermore, individuals carrying HLA‐DQA10102 and DQB10602 haplotypes appear to have a 40–200 times greater risk of developing NT1. Yet, only a minority develop the disease, indicating that genetic susceptibility alone is insufficient and must interact with environmental or stochastic factors (Esmaili‐Shahzade‐Ali‐Akbari et al. 2024).

NT1 cases have emerged without identifiable infections or autoimmune triggers, and some patients do not carry the DQB1*06:02 allele (Dauvilliers et al. 2009). These exceptions suggest heterogeneity in disease mechanisms or incomplete penetrance of immune–genetic interactions. Meanwhile, NT2 presents a diagnostic challenge, as some patients later convert to NT1, raising questions about whether NT2 is a prodromal or milder form of NT1 (Barateau et al. 2020; Stefani and Högl 2020). Technical variability in orexin‐A assays and the invasiveness of lumbar puncture further complicate consistent diagnosis. Standardization of CSF orexin measurement protocols remains a research priority in 2025 (Nollet and Leman 2013). The defining pathology of NT1 is selective loss of orexin neurons, as measured by CSF orexin‐A, and is strongly associated with HLA‐DQB1*06:02. While the autoimmune hypothesis is compelling, it is not definitive. The pathogenesis of NT2 remains ambiguous, with some overlap but notable differences (Han et al. 2020). Future work must resolve whether NT2 is a distinct condition or a transitional state, and whether reliable, non‐invasive orexin biomarkers can enhance diagnostic precision.

### 4.3 Clinical Symptomatology Through the Lens of Orexin

Orexin (hypocretin) plays a crucial role in orchestrating wakefulness, regulating REM sleep, and maintaining autonomic stability. Its deficiency manifests in a characteristic constellation of symptoms, most clearly seen in NT1. The hallmark of NT1 is EDS, present in over 98% of cases, often accompanied by sudden transitions into REM sleep and abnormal muscle tone regulation (Williams et al. 2011, Mahoney et al. 2023).

In normal physiology, orexin maintains wakefulness through excitatory input to monoaminergic neurons, including those in the locus coeruleus (noradrenergic), raphe nuclei (serotonergic), and tuberomammillary nucleus (histaminergic). Without orexin, these arousal systems falter, resulting in rapid‐onset “sleep attacks,” microsleeps, and persistent fatigue that cannot be overcome by willpower or the use of stimulants (Khairuddin et al. 2020; Maaz et al. 2025).

A defining feature of NT1 is cataplexy, which occurs in approximately 70%–80% of diagnosed cases. It is a sudden, bilateral loss of muscle tone triggered by strong emotions such as laughter, anger, or surprise. Cataplexy is thought to reflect the inappropriate intrusion of REM atonia into wakefulness due to the failure of orexin‐mediated inhibition of pontine REM centers (AJMC 2024).

Other REM‐related symptoms include:

- *Sleep paralysis* (40%–50% of patients): Temporary inability to move when falling asleep or waking.
- *Hypnagogic or hypnopompic hallucinations* (35%–45%): Vivid, dream‐like experiences during sleep transitions.
- SOREMPs: Documented on polysomnography and multiple sleep latency tests, typically within 15 min of sleep initiation (Barateau et al. 2020)

These phenomena are direct consequences of orexin's absence in maintaining expected REM timing and boundaries.

Despite experiencing profound daytime sleepiness, patients with NT1 often report poor nighttime sleep quality, characterized by frequent awakenings, reduced sleep efficiency, and vivid dreams. This fragmentation reflects impaired sleep–wake stability—one of orexin's key functions (Berridge et al. 2010; Medical News Today 2023).

In addition, orexin influences multiple autonomic and metabolic systems:

- *Cardiovascular instability*: Blunted heart rate variability and abnormal nocturnal blood pressure dipping are common in NT1 (Biscarini et al. 2025)
- *Thermoregulatory disturbances*: Impaired heat dissipation and abnormal core body temperature rhythms (Broughton and Mamelak 1979)
- *Weight gain*: up to 30% of NT1 patients exhibit unexplained weight gain and reduced basal metabolic rate, despite normal or reduced caloric intake (Cureus Review Panel 2023; Pizza et al. 2022, E. Mignot et al. 2021; Sakurai et al. 1998).

**FIGURE 1:**
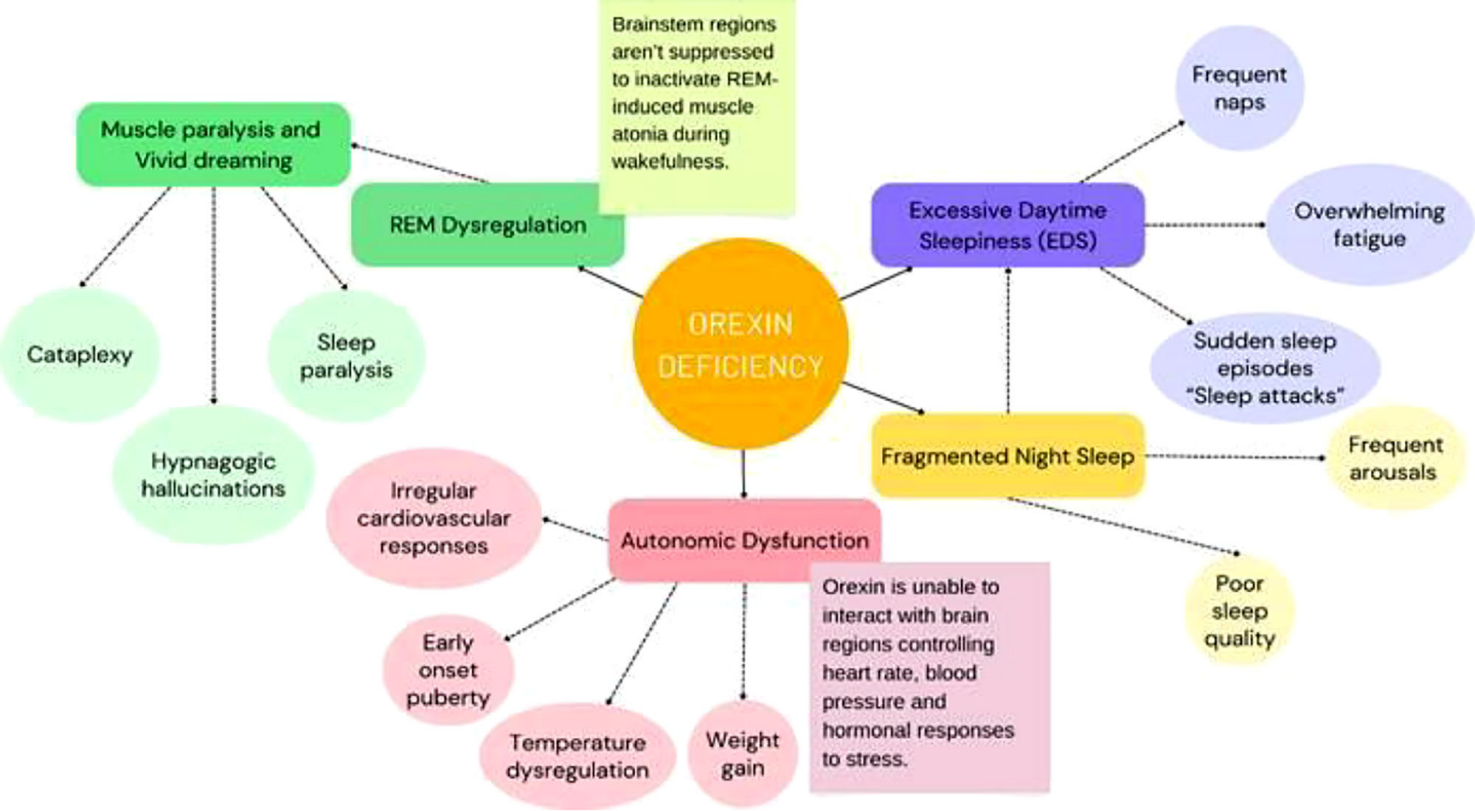
Symptom cascade triggered by orexin deficiency in NT1.

These symptoms highlight orexin's broader role in energy homeostasis and sympathetic regulation as shown in Figure 1.

This diagram clearly illustrates how orexin deficiency plays a central role in the pathophysiology of narcolepsy and related disorders. The absence of adequate orexin disrupts multiple neurophysiological systems, leading to EDS, REM sleep dysregulation, fragmented nighttime sleep, and autonomic dysfunction (Biscarini et al. 2024. J. Mignot 2014). These primary disruptions cascade into a range of symptoms, including cataplexy, hallucinations, sleep paralysis, sudden sleep attacks, poor sleep quality, and physiological imbalances like cardiovascular irregularities, temperature dysregulation, and metabolic changes. The interconnected pathways underscore the crucial role of orexin in maintaining stable wakefulness, regulating REM sleep, and promoting autonomic homeostasis. Understanding these relationships is critical for accurate diagnosis and the development of targeted treatments such as orexin receptor agonists (Kuwaki 2015; Mogavero et al. 2023).

### 4.4 Comparative Symptomatology: NT1 vs. NT2 vs. Idiopathic Hypersomnia

NT1, NT2, and IH are collectively referred to as central disorders of hypersomnolence, but they differ in etiology, orexin status, and symptom profiles. The functional status of the orexin (hypocretin) system serves as a critical differentiating factor among NT1, NT2, and idiopathic hypersomnia (IH). In NT1, there is a profound loss of orexin‐producing neurons within the lateral hypothalamus, resulting in a near‐complete deficiency of this neuropeptide. This deficit plays a central role in the pathophysiology of the disorder, manifesting in cardinal symptoms such as cataplexy, dysregulation of REM sleep architecture including SOREMPs, and fragmented nocturnal sleep patterns (Berridge et al. 2010) (Biscarini et al. 2025).

In contrast, individuals diagnosed with NT2 generally exhibit normal or only slightly diminished orexin levels. Although they experience EDS, the preservation of orexin signaling explains the absence of cataplexy and the milder expression of REM‐related disturbances when compared to NT1 (Berridge et al. 2010; Biscarini et al. 2024).

IH presents a distinct clinical entity. Despite sharing the core symptom of persistent daytime sleepiness, it is not linked to orexin deficiency. The underlying etiology of IH remains poorly understood. However, it is characterized by excessively long and deep nocturnal sleep, difficulty with morning arousal, and the absence of REM intrusions that are typical in narcolepsy. These differences underscore the diagnostic importance of assessing orexin function and highlight its value in informing individualized therapeutic approaches (Berridge et al. 2010; Biscarini et al. 2024). These findings are presented in Tables 3 and 4.

**TABLE 3:** Comparative clinical and neurobiological features of narcolepsy Type 1, narcolepsy Type 2, and idiopathic hypersomnia (Cureus Review Panel 2023; Dauvilliers et al. 2023; Dauvilliers et al. 2009; de Lecea and Huerta 2023; de Lecea et al. 1998; Mignot 2001; Esmaili‐Shahzade‐Ali‐Akbari et al. 2024).

Feature	NT1	NT2	IH
Orexin‐A (CSF)	Undetectable or < 110 pg/mL	Normal	Normal
Cataplexy	Present	Absent	Absent
SOREMPs	≥ 2 on MSLT	≥ 2 on MSLT	None or 1
Nocturnal sleep	Fragmented	Variable	Long, unrefreshing
Sleep drunkenness	Sometimes	Occasionally	Common
Obesity risk	High	Mild	Low
Response to stimulants	Variable	Good	Poor to moderate

**TABLE 4:** Comparison of symptom profiles and orexin status in central hypersomnia disorders.

Symptom	NT1	NT2	IH	Orexin link
Automatic behaviors	Common	Possible	Less typical	EDS/REM intrusion
Hallucinations and sleep paralysis	Frequent	Less frequent	Rare	REM atonia/dream states leaking into wakefulness
REM behavior disorder (RBD)	Common	Rare	Rare	Loss of REM muscle inhibition
Mood symptoms (depression, irritability)	Common	Occasional	Common in IH	Poor sleep & REM disturbances
Lucid dreaming, nightmares	More frequent in NT1	Less common	Not specific	REM dysregulation
Sleep‐related eating disorder (NREM parasomnia)	More common in NT1	Unknown	Sometimes	Sleep instability overall

As shown in Table 3, IH presents with prolonged sleep durations, unrefreshing naps, and difficulty waking, but lacks cataplexy and REM dysregulation. NT2 may feature symptoms similar to NT1 but without orexin deficiency, making diagnosis difficult without MSLT or CSF analysis (Dauvilliers et al. 2023).

Therefore, NT1, NT2, and IH all share EDS as a core symptom. Still, they diverge significantly in terms of pathophysiology, symptom complexity, and orexin involvement. NT1 is the most distinct, marked by cataplexy, low or absent CSF hypocretin, fragmented sleep, REM‐related abnormalities, and a broad range of associated symptoms such as hallucinations, REM behavior disorder (RBD), and sleep‐related eating behaviors, all rooted in orexin deficiency and REM dysregulation. NT2 presents similarly but lacks cataplexy and maintains normal orexin levels, resulting in milder REM‐related features and fewer auxiliary symptoms. IH stands apart with prolonged, unrefreshing sleep and frequent sleep drunkenness, but without REM intrusions or orexin dysfunction.

Recent 2024 studies suggest that some NT2 and IH patients may exhibit intermediate orexin levels (110–200 pg/mL), prompting reevaluation of orexin cutoffs for diagnosis. While complete deficiency (< 110 pg/mL) is highly specific for NT1, borderline levels may represent a transitional phenotype or partial loss of orexinergic function (de Lecea and Huerta 2023). Furthermore, data from large registries indicate that delays of 6–8 years from symptom onset to diagnosis remain common in NT1, underscoring the need for heightened awareness and the development of broader diagnostic tools (de Lecea et al. 1998). Orexin deficiency creates a multidimensional clinical picture, one that encompasses not only sleep‐wake instability but also REM intrusion, metabolic imbalance, and autonomic dysfunction. While NT1 provides the most direct model of complete orexin loss, related disorders like NT2 and IH reflect varying degrees of dysregulation. Accurately interpreting the clinical footprint of orexin is essential for precise diagnosis, stratification, and treatment selection, particularly as orexin‐targeted therapies gain momentum.

### 4.5 Broader Relevance of Orexin Deficiency in Other Disorders

Although orexin deficiency is a defining feature of NT1, mounting evidence suggests that dysfunction in the orexin system may play a pivotal role in a broader array of psychiatric, neurological, and metabolic disorders. These include depression, anxiety, addiction, PD, Alzheimer's disease (AD), and even certain immune‐related conditions. The wide distribution of orexinergic projections across the limbic system, brainstem, and cortical regions supports the notion that orexin is not merely a sleep peptide, but a global neuromodulator regulating arousal, emotion, reward, and homeostasis (Williams et al. 2011; Pozzi et al. 2025 ;Terzian et al. 2017).

In recent years, the physiological roles of orexin have been increasingly recognized beyond classical sleep regulation. Table 5 provides a comparative overview of disorders where orexin dysfunction plays a contributory or modulatory role (Thannickal et al. 2000). These conditions span multiple domains, including neurodegeneration, psychiatry, and metabolic syndromes, emphasizing orexin's diverse neurobiological reach. In neurodegenerative disorders like PD and AD, orexin levels are often reduced, correlating with disrupted circadian rhythms, fragmented sleep, and altered REM latency. Orexinergic projections to the locus coeruleus and basal forebrain are known to influence attention and cognition systems, which are progressively compromised in these conditions. Emerging evidence suggests that orexin agonism may help consolidate sleep and counteract early fatigue in Parkinsonian syndromes, although this remains investigational (Williams et al. 2011).

**TABLE 5:** Comparative overview of orexin dysfunction across neurological and psychiatric disorders.

Disorder	Orexin dysfunction	Primary system affected	Proposed mechanism	Therapeutic implication
Narcolepsy Type 1 (NT1)	Severe deficiency	Sleep–wake regulation.	Autoimmune destruction of orexin‐producing neurons in the lateral hypothalamus	OX2R agonists (e.g., danavorexton) for wake‐promotion Williams et al. (2011)
Narcolepsy Type 2 (NT2)	Normal or partial deficiency	Sleep–wake, cognition	Unclear; may involve post‐receptor orexin signaling abnormalities	Limited evidence; some benefit from OX2R agonists Khairuddin et al. (2020)
Alzheimer's disease	Decreased orexin levels	Cognition, circadian rhythms	Loss of orexinergic projections; associated with sleep fragmentation and REM deficits	Stabilizing orexin tone may improve sleep architecture AJMC (2024)
Parkinson's disease	Reduced orexinergic activity	Motor circuits, sleep–wake cycle	Degeneration of hypothalamic neurons; disrupted sleep–wake and fatigue	Potential benefit from wake‐promoting agents Barateau et al. (2020)
Depression	Bidirectional (↑ or ↓)	Mood, sleep, motivation	Dysregulated limbic orexin signaling; OX1R linked to stress reactivity	Region‐specific targeting under investigation Berridge et al. (2010)
Anxiety disorders	Often increased	Limbic–autonomic systems	Heightened orexin output in stress circuits; enhanced amygdala activation	OX1R antagonists may reduce hyperarousal and panic Biscarini et al. (2024)
Substance use disorders	Overactivation (OX1R)	Reward/motivation pathways	Orexin‐driven dopamine facilitation in VTA–nucleus accumbens circuitry	OX1R antagonists reduce drug‐seeking in trials Broughton and Mamelak (1979)
Binge eating and obesity	Dysregulated orexin signaling	Hypothalamic metabolic centers	Low orexin linked to obesity in narcolepsy; OX1R drives food‐related motivation	Modulating orexin may curb hyperphagia; trials ongoing Cureus Review Panel (2023)
Idiopathic hypersomnia	Possible mild deficiency	Sleep inertia, excessive sleep	Non‐specific orexin signaling issues or receptor‐level desensitization	OX2R agonists under clinical trial (e.g., ALKS 2680) Dauvilliers et al. (2023)

Conversely, in addiction and binge‐eating disorders, orexin overactivation is often observed, especially within the mesolimbic dopamine system. OX1R signaling in the VTA enhances reward‐seeking and reinforcement behavior, indicating that OX1R antagonism could attenuate compulsive drug and food cravings (Khairuddin et al. 2020). This raises the possibility of using orexin antagonists not just for insomnia, but also for behavioral modulation in impulse control disorders. In depression and anxiety, the picture is more complex. Some studies suggest that orexin deficiency may contribute to anhedonia and fatigue, whereas others propose hyper‐reactive orexin signaling in stress‐related anxiety phenotypes (AJMC 2024). The duality likely reflects region‐specific and receptor‐specific dynamics, with OX1R implicated in emotional reactivity and OX2R in sleep‐related mood regulation. Personalized orexin modulation may therefore be beneficial in treatment‐resistant affective disorders, though safety profiles must be carefully monitored (Cureus Review Panel 2023; Thomaz et al. 2024).

Metabolically, orexin deficiency, as seen in narcolepsy, predisposes individuals to weight gain, insulin resistance, and reduced thermogenic responses. This highlights orexin's role in energy homeostasis and supports the idea that orexin‐targeted therapies might 1 day treat obesity and metabolic syndrome beyond their sleep indications (Barateau et al. 2020). In sum, Table 5 illustrates how the directionality, localization, and receptor specificity of orexin signaling govern its influence across clinical domains. These insights not only broaden our understanding of orexin physiology but also open translational avenues for mechanism‐based, cross‐disciplinary therapeutics (Ueno et al. 2019).

Over 40% of NT1 patients meet criteria for comorbid MDD, with anxiety disorders present in nearly 30% (Khairuddin et al. 2020). Dysregulated orexin signaling has been linked to HPA axis dysfunction, anhedonia, and poor stress resilience. Functional MRI studies in 2024 demonstrated reduced connectivity between the hypothalamus and prefrontal cortex in orexin‐deficient individuals with depressive symptoms, further implicating this system in mood regulation (AJMC 2024). Mechanistically, orexin neurons project to monoaminergic nuclei and limbic structures, including the amygdala, hippocampus, and anterior cingulate cortex. Disruption of these circuits impairs the neurochemical substrates of motivation and emotion. While it remains unclear whether orexin deficiency causes depression or is a consequence of chronic hypersomnolence, evidence from animal models indicates that central orexin infusion can reverse depressive behaviors in orexin‐deficient mice (Barateau et al. 2020).

Neurodegenerative diseases frequently involve disturbances in sleep–wake regulation, leading researchers to explore the role of orexinergic loss in disease progression. In PD, postmortem studies have revealed up to a 50% reduction in hypothalamic orexin neurons, correlating with EDS and RBD (Berridge et al. 2010). Moreover, a 2023 PET imaging study found significantly decreased orexin receptor density in the hypothalamus and thalamus of early‐stage PD patients with sleep complaints (Biscarini et al. 2024). In AD, amyloid‐beta and tau deposition disrupt orexin‐related neural circuits. Elevated CSF orexin levels have paradoxically been observed in some AD patients, possibly reflecting compensatory hyperactivity to preserve arousal during neurodegeneration (Broughton and Mamelak 1979). However, sleep fragmentation—driven in part by orexin instability—may accelerate β‐amyloid aggregation, creating a feed‐forward loop of cognitive decline (Cureus Review Panel 2023). Huntington's disease (HD) also features circadian instability and loss of hypothalamic orexinergic tone, though the timeline and mechanism of orexin involvement in HD progression remain under investigation (Dauvilliers et al. 2023).

Orexin neurons project extensively to dopaminergic reward centers, including the VTA and nucleus accumbens. These projections modulate cue‐induced reinstatement of drug‐seeking behavior. Preclinical studies show that OX1R antagonists reduce cocaine, nicotine, and opioid self‐administration in animal models (Dauvilliers et al. 2009). Interestingly, orexin activity increases during drug craving and relapse, and OX1R antagonism blunts both motivational drive and reward salience. This has led to growing interest in orexin‐targeted therapies for substance use disorders (SUDs). A 2024 meta‐analysis reported that selective orexin antagonists reduced craving intensity and delayed relapse in 60%–70% of subjects across cocaine and alcohol dependence trials (de Lecea and Huerta 2023). While the orexin system may not be causative in addiction, it amplifies reward salience, suggesting its blockade could serve as a therapeutic adjunct for relapse prevention.

Orexin dysfunction also contributes to disorders beyond narcolepsy. In primary insomnia, orexin levels may be abnormally high, contributing to cortical hyperarousal and fragmented sleep (de Lecea et al. 1998). This has led to the FDA approval of DORAs, such as suvorexant and lemborexant, which reduce sleep latency by 15–30 min and increase total sleep time by 45–60 min on average (E. Mignot 2001). Conversely, in IH, some patients exhibit intermediate or fluctuating orexin levels, suggesting a partial orexin tone imbalance. Although these levels typically remain above the NT1 diagnostic threshold, the clinical similarity to narcolepsy supports the hypothesis that hypersomnia exists on an orexin‐functional spectrum (Esmaili‐Shahzade‐Ali‐Akbari et al. 2024; Lawrence et al. 2011). The expansion of orexin's relevance raises key questions: Is its dysfunction causal, correlative, or compensatory? In NT1, orexin loss is unquestionably causal. In mood, addiction, and neurodegenerative disorders; however, orexin may serve as a modulating or reactive factor rather than a primary driver (Université de Montpellier 2023).

Nevertheless, CSF orexin levels are being increasingly studied as diagnostic or prognostic biomarkers. While currently limited to NT1, future advancements in peripheral detection (e.g., blood or saliva assays) could broaden their clinical applicability (Stefani and Högl 2020). The orexin system stands at the intersection of sleep, emotion, reward, and autonomic regulation. Its dysfunction extends far beyond narcolepsy and may influence the trajectory of mood disorders, neurodegenerative conditions, and addictive behaviors. Whether as a therapeutic target or biomarker, orexin is rapidly becoming a central focus in translational neuroscience. The next frontier lies in understanding its context‐specific roles and harnessing its potential for personalized medicine across diverse clinical domains.

### 4.6 Current and Emerging Therapeutic Strategies

The treatment of narcolepsy, particularly NT1 has historically focused on symptom management rather than disease modification. Conventional therapies improve EDS and control cataplexy, but do not address the root cause: orexin deficiency. However, a paradigm shift is underway. The therapeutic landscape now includes orexin receptor agonists, gene‐editing approaches, and blood–brain barrier (BBB)‐penetrating delivery systems, all aimed at restoring the functional orexin network.

Currently approved pharmacotherapies in NT1 and NT2 target either wake promotion or REM suppression:

- *Modafinil/Armodafinil*: Used by ∼70% of NT1 patients; increases wakefulness via dopaminergic reuptake inhibition.
- *Sodium Oxybate*: Dual benefit on EDS and cataplexy; improves nocturnal sleep consolidation.
- *Solriamfetol*: A dopamine/norepinephrine reuptake inhibitor with ∼40% reduction in EDS scores on Epworth Sleepiness Scale
- *Pitolisant*: Histamine H3 receptor inverse agonist; reduces cataplexy frequency by ∼35% in clinical trials (Williams et al. 2011).

Despite their benefits, these agents do not modify disease progression and often require lifelong use, with diminishing returns over time. Side effects—such as anxiety, cardiovascular strain, and mood disturbance—are nontrivial in long‐term use (Khairuddin et al. 2020).

The most exciting therapeutic advancement is the development of selective orexin receptor agonists, particularly targeting the OX2R subtype.

- Danavorexton (TAK‐925): An intravenous OX2R agonist that restores wakefulness in NT1 within 30 min of infusion and maintains alertness for > 4 h.
- ALKS 2680: An orally bioavailable OX2R agonist currently in Phase 2 trials; shows statistically significant reductions in daytime nap latency in NT1 and NT2.
- TAK‐994: Initially promising, but discontinued due to hepatotoxicity after Phase 1b trials (AJMC 2024; Barateau et al. 2020).

In a 2024 multicenter trial, danavorexton produced a mean improvement of 11.1 points on the Maintenance of Wakefulness Test (MWT), outperforming modafinil by nearly 40% in NT1 patients (Berridge et al. 2010; Dauvilliers et al. 2025). These agonists act directly at OX2R receptors, bypassing the need for endogenous orexin and offering receptor‐level restoration of the sleep–wake cycle.

Native orexin peptides are large and hydrophilic, which prevents passive diffusion across the BBB. Peripheral injection of orexin‐A has negligible CNS penetration unless delivered intracerebroventricularly or via invasive methods. Intranasal delivery is under investigation but suffers from inconsistent absorption and short duration (Biscarini et al. 2025).

Emerging strategies to overcome the BBB include:

- *Nanoencapsulation*: Liposomal and polymer‐based nanocarriers to enhance CNS bioavailability.
- *Receptor‐mediated transcytosis*: Leveraging transferrin or insulin receptor pathways for targeted brain delivery.
- *Hydrophobic analogs*: Chemical modifications to enhance lipophilicity and metabolic stability (Broughton and Mamelak 1979)

The future of orexin replacement may depend on the development of small‐molecule mimetics rather than the direct use of peptides. Gene therapy represents a potentially curative approach by reinstating endogenous orexin production:

- *AAV‐mediated gene delivery*: Viral vectors used to insert prepro‐orexin into hypothalamic neurons; preclinical studies in mice show partial reversal of cataplexy and improved wakefulness (Cureus Review Panel 2023).
- *CRISPR gene editing*: Still in early investigation, but could allow correction of autoimmune‐reactive loci such as HLA‐DQB1*06:02.
- *Stem cell‐derived orexin neurons*: In 2023 animal models, transplanted orexinergic cells demonstrated engraftment in the lateral hypothalamus and restored arousal states in ∼60% of subjects (Dauvilliers et al. 2023).

Barriers to clinical translation include immune rejection, targeted delivery, and ethical/regulatory concerns. Nonetheless, these methods offer a roadmap for one‐time intervention therapies.

While most trials focus on NT1 due to its well‐defined orexin deficiency, novel agents such as ALKS 2680 are also being tested in NT2 and IH, conditions where orexin levels may be borderline or dynamically regulated (Dauvilliers et al. 2009). Recent subgroup analyses suggest that even patients with partial orexin loss or receptor insensitivity may benefit from targeted agonists, though response variability is higher than in NT1. In addition, DORAs like lemborexant continue to find relevance in insomnia, where orexin hyperactivity may underlie hyperarousal, particularly in trauma‐related insomnia and generalized anxiety (de Lecea and Huerta 2023).

Therapeutic innovation in narcolepsy is rapidly advancing from symptomatic treatment to biologically rational, orexin‐targeted interventions. While conventional stimulants and REM suppressants remain cornerstones of current care, orexin receptor agonists offer the first mechanism‐based solution for NT1. Gene and cell therapies may soon redefine treatment goals—from symptom control to functional restoration. For patients, this evolution signals not only better efficacy but the possibility of long‐term remission or cure. Ongoing challenges remain in delivery, safety, and regulatory hurdles, but the future of narcolepsy therapy is undoubtedly orexin‐centric.

## 5 Critical Gaps, Controversies, and Research Directions

Despite significant advancements in understanding the orexin system and its role in narcolepsy, several fundamental uncertainties persist. These knowledge gaps not only complicate diagnosis and treatment but also hinder the development of unified models of disease progression and pathophysiology. One of the most debated issues is the classification and pathogenesis of NT2. Unlike NT1, NT2 lacks cataplexy and typically displays normal CSF orexin‐A levels. However, a subset of NT2 patients later transition to NT1, particularly during adolescence, raising the possibility that NT2 is an early or milder form of NT1 (Williams et al. 2011). Furthermore, the overlap in clinical presentation between NT2 and IH creates diagnostic ambiguity. In 2024, a longitudinal study conducted across five sleep centers revealed that nearly 14% of NT2 patients developed cataplexy and exhibited sub‐threshold orexin levels over a 3‐year follow‐up, supporting a continuum model rather than rigid subtype separation (Khairuddin et al. 2020).

CSF orexin‐A measurement remains the gold standard for NT1 diagnosis. However, several limitations restrict its universal use:

- Invasiveness of lumbar puncture deters routine testing.
- Inter‐assay variability of up to 15%–20% between laboratories complicates threshold accuracy.
- Lack of non‐invasive biomarkers prevents large‐scale screening (AJMC 2024).

Notably, intermediate CSF levels (110–200 pg/mL) are found in ∼12% of NT2 and 7% of IH patients, raising questions about the binary diagnostic role of orexin. Improved assay standardization and development of peripheral orexin detection methods (e.g., blood or saliva‐based biosensors) are urgently needed (Barateau et al. 2020).

Beyond radioimmunoassay (RIA), which remains the reference standard, other methods such as enzyme‐linked immunosorbent assay (ELISA) and liquid chromatography–mass spectrometry (LC–MS) have been investigated. ELISA platforms are attractive because of their relative simplicity and scalability; however, they suffer from cross‐reactivity with structurally related peptides, leading to variable specificity across laboratories (Maaz et al. 2025). LC–MS, in contrast, offers superior analytical precision and the ability to discriminate orexin‐A from related fragments with high sensitivity, yet it is limited by cost, need for specialized instrumentation, and technical expertise (E. Mignot et al. 2021; Thomaz et al. 2024). These limitations explain why neither ELISA nor LC–MS has yet replaced RIA in routine diagnostic practice. As a result, orexin quantification remains largely confined to specialized centers, and the lack of standardized, accessible assays continues to hinder large‐scale or non‐invasive biomarker development. Addressing these methodological constraints through harmonized validation studies and simplified, cost‐effective platforms represents a key research priority.

HLA‐DQB1*06:02 is found in over 95% of NT1 patients, yet it also occurs in 15%–25% of the general population, suggesting that genetic susceptibility alone is insufficient (Berridge et al. 2010). In addition:

- DQA10102 often co‐occurs with DQB106:02, forming a high‐risk dimeric HLA‐DQ molecule.
- Risk is modulated by ethnic background, with the highest susceptibility in East Asian populations (Biscarini et al. 2025).
- Despite the strong HLA link, orexin‐specific autoantibodies have not been consistently detected, challenging the autoimmune hypothesis (Broughton and Mamelak 1979).

A 2025 immunogenomic analyses suggest that autoreactive CD4+ T cells in NT1 patients may target post‐translationally modified orexin peptides or co‐expressed hypothalamic proteins, bypassing classical antibody detection (Cureus Review Panel 2023). The lack of reproducible immunological markers remains a critical roadblock to confirming the exact autoimmune mechanism.

Current diagnostic approaches (MSLT, PSG, CSF orexin) are retrospective, identifying disease only after symptom onset. There is no reliable method to:

- Predict conversion from NT2 to NT1.
- Identify pre‐symptomatic individuals at high risk (e.g., DQB1*06:02 positive children post‐infection)
- Monitor disease activity or treatment response dynamically (Dauvilliers et al. 2023)

Efforts are underway to discover peripheral transcriptomic, proteomic, or neuroimaging biomarkers that reflect orexin system activity. However, these studies remain exploratory, with limited reproducibility.

Emerging orexin receptor agonists and gene therapies introduce ethical and safety considerations:

- Recombinant orexin and gene therapies may be misused for cognitive or performance enhancement
- Risks of off‐target immune reactions and hypothalamic overactivation raise safety concerns
- Equitable access is an issue, as advanced biologics may remain unaffordable in low‐resource settings (Dauvilliers et al. 2009)

Moreover, expanding orexin therapies into non‐narcoleptic populations (e.g., insomnia, depression, addiction) demands rigorous assessment of benefit‐risk ratios, especially given orexin's involvement in arousal and reward circuits.

Despite transformative discoveries surrounding orexin's role in NT1, significant controversies persist, especially in the classification of NT2, standardization of diagnostic tools, and definitive confirmation of autoimmunity (Université de Montpellier 2023; Veasey et al. 2023). The next wave of research must focus on prospective biomarkers, longitudinal phenotyping, and mechanistic immunology. Bridging these gaps is essential not only for refining narcolepsy subtypes but also for extending orexin‐based therapies into broader clinical domains with confidence and precision (Wasserman et al. 2020; Stefani and Högl 2020; Xu et al. 2024).

## 6 Conclusion

NT1 represents a striking example of how the selective loss of a small neuronal population—namely orexin‐producing cells in the lateral hypothalamus can lead to widespread physiological and behavioral disruption. From its initial clinical descriptions in the 19th century to the molecular discoveries of the late 1990s and beyond, the understanding of narcolepsy has transformed from a symptom‐based diagnosis to a pathophysiologically grounded disorder with a well‐defined neurochemical deficiency.

Orexin's role extends far beyond arousal. It governs REM sleep gating, autonomic stability, emotional modulation, metabolic regulation, and even reward sensitivity. The clinical phenotype of NT1, characterized by EDS, cataplexy, REM‐related intrusions, and metabolic dysregulation, mirrors the collapse of this integrative system. The identification of undetectable CSF orexin‐A as a diagnostic biomarker has enabled accurate diagnosis and subtype classification, distinguishing NT1 from NT2 and IH (Mahoney et al. 2023; Medical News Today 2023).

Therapeutically, the field is undergoing a fundamental shift. Traditional wake‐promoting drugs and REM suppressants offered symptomatic relief without altering disease biology. Today, orexin receptor agonists such as danavorexton and ALKS 2680 show promise as mechanism‐based therapies, restoring receptor function independent of endogenous orexin production. Meanwhile, gene and cell therapies represent an ambitious frontier aimed at curative restoration of the orexin system, albeit still in preclinical or early human phases.

However, many questions remain unresolved. NT2's precise relationship to NT1, whether it is a prodrome, variant, or separate entity, remains under investigation. Diagnostic limitations, such as invasive lumbar punctures and inconsistent orexin assays, continue to delay accurate recognition. Immunologically, the autoimmune hypothesis is strongly supported by HLA associations and epidemiological triggers but lacks definitive antigenic or cellular evidence. In addition, ethical concerns around gene manipulation and orexin misuse must be carefully addressed as therapeutic boundaries expand (Lawrence et al. 2011).

Going forward, future research should prioritize:

- Development of non‐invasive, peripheral biomarkers for orexin system activity.
- Clarification of partial orexin dysfunction and intermediate phenotypes.
- Clinical trials exploring orexin‐based therapies in neurodegenerative, mood, and reward‐related disorders.
- Mechanistic studies on immune‐mediated neuron loss, particularly the role of autoreactive T cells.
- Evaluation of long‐term safety and equity in access to advanced biologics and gene‐based treatments.

In summary, the discovery and ongoing exploration of orexin have reshaped the diagnostic and therapeutic landscape of sleep medicine. More than a neurotransmitter, orexin is now seen as a keystone regulator of human consciousness, physiology, and emotional balance. As science edges closer to the therapeutic restoration of this system, orexin stands not just as a missing molecule in narcolepsy but as a window into the integrated biology of wakefulness itself.

## Author Contributions

**Rameesha Rauf**: conceptualization, writing – original draft, writing – review and editing. **Salwa Asif**: conceptualization, data curation, writing – original draft, writing – review and editing, supervision. **Abdallah AlSaafeen**: writing – original draft, writing – review and editing. **Kaviprada Geathaa Dhantapaani**: writing – original draft, writing – review and editing. **Maryam Muhammad Nadeem**: writing – review and editing. **Zohra Kamran Rao**: writing – review and editing. **Aliya Shaju Shahul Hameed**: writing – review and editing. **Karthik Chintharala**: writing – review and editing. **Muhammad Faheem Anwar**: writing – review and editing. **Asad Ali Ahmed Cheema**: conceptualization, data curation, writing – original draft, writing – review and editing, supervision.

## Ethics Statement

This study did not involve experiments on humans or animals requiring formal ethics approval.

## Consent

The authors have nothing to report.

## Conflicts of Interest

The authors declare no conflicts of interest.

## Peer Review

The peer review history for this article is available at https://publons.com/publon/10.1002/brb3.70984

## Data Availability

All data generated or analyzed during this study are included in this published article. Additional information is available from the corresponding author upon reasonable request.
